# Evaluation of capillary density in psoriasis: An intrapatient study and literature review

**DOI:** 10.1371/journal.pone.0247835

**Published:** 2021-03-10

**Authors:** Giuseppe Micali, Anna Elisa Verzì, Giuseppe Broggi, Rosario Caltabiano, Maria Letizia Musumeci, Francesco Lacarrubba

**Affiliations:** 1 Dermatology Clinic, University of Catania, Catania, Italy; 2 Department "G.F. Ingrassia", Section of Anatomic Pathology, University of Catania, Catania, Italy; Northwestern University Feinberg School of Medicine Galter Health Sciences Library, UNITED STATES

## Abstract

**Background:**

Dilated and tortuous vessels within elongated dermal papillae represent a histopathological clue of psoriasis. However, the number of dilated capillaries (capillary density) in psoriasis remains undefined as the results from the available studies differ significantly.

**Objectives:**

To evaluate the capillary density in psoriasis using dermoscopy and horizontal histopathological sections (HHS), two techniques that share the horizontal view of the skin, and to compare the results with the existing data.

**Methods:**

Twenty adult patients with stable plaque psoriasis were enrolled and, in each patient, a target area of the examined plaque, previously engraved by gently rotating a 5-mm biopsy punch device, underwent dermoscopy and biopsy for HHS. In all examined fields, capillary density was evaluated in a centered 4-mm diameter area, counting the number of red dots at dermoscopy and of dermal papillae at HHS.

**Results:**

A total of 20 target lesions located on the trunk, arms and tights were evaluated. The mean capillary density resulting from dermoscopy was 43.02±6.60/mm 2 whereas that from HHS was 50.30±9.05/mm 2. These data showed a statistically significant difference (p = 0.006), with a strong correlation at Pearson’s test (r = 0.88).

**Conclusions:**

Our results when compared with those from the existing literature showed some differences. The peculiarity of our work is represented by the precise measurement and correlation of the capillary density using two different methods, as the preliminary skin engraving allowed a perfect match between the area undergoing dermoscopy and that of skin sampling for HHS. Compared to dermoscopy in which deep-located vessels might have gone undetected, HHS seems to reflect more precisely and reliably the real capillary density showing an average of 50 capillaries/mm 2 that in a common 5x5 cm psoriatic patch corresponds to an average of 125.000 capillaries. These results highlight the extraordinary potential of psoriatic skin to develop such a complex and intricate vascular network.

## Introduction

Psoriasis is a common skin disease whose complex pathogenetic mechanisms including vascular remodeling have recently been demonstrated. Dilated and tortuous vessels within elongated dermal papillae represent a histopathological clue also evident by dermoscopy, a non-invasive technique that magnifies (X10) the skin surface, as uniformly distributed “red dots” [1, 2]. A recent study using horizontal histopathological sections (HHS) on psoriatic skin confirmed the presence of roundish dermal papillae at the dermo-epidermal junction each centered by ectatic capillaries [3]. However, the number of dilated capillaries (i.e. capillary density) in psoriasis remains undefined as the results from the available studies differ significantly [4–13].

The objectives of this study were to evaluate the capillary density in psoriasis using HHS and to compare the results with those obtained using dermoscopy, also sharing the horizontal view of the skin, and with the existing data.

## Materials and methods

**Fig 1:**
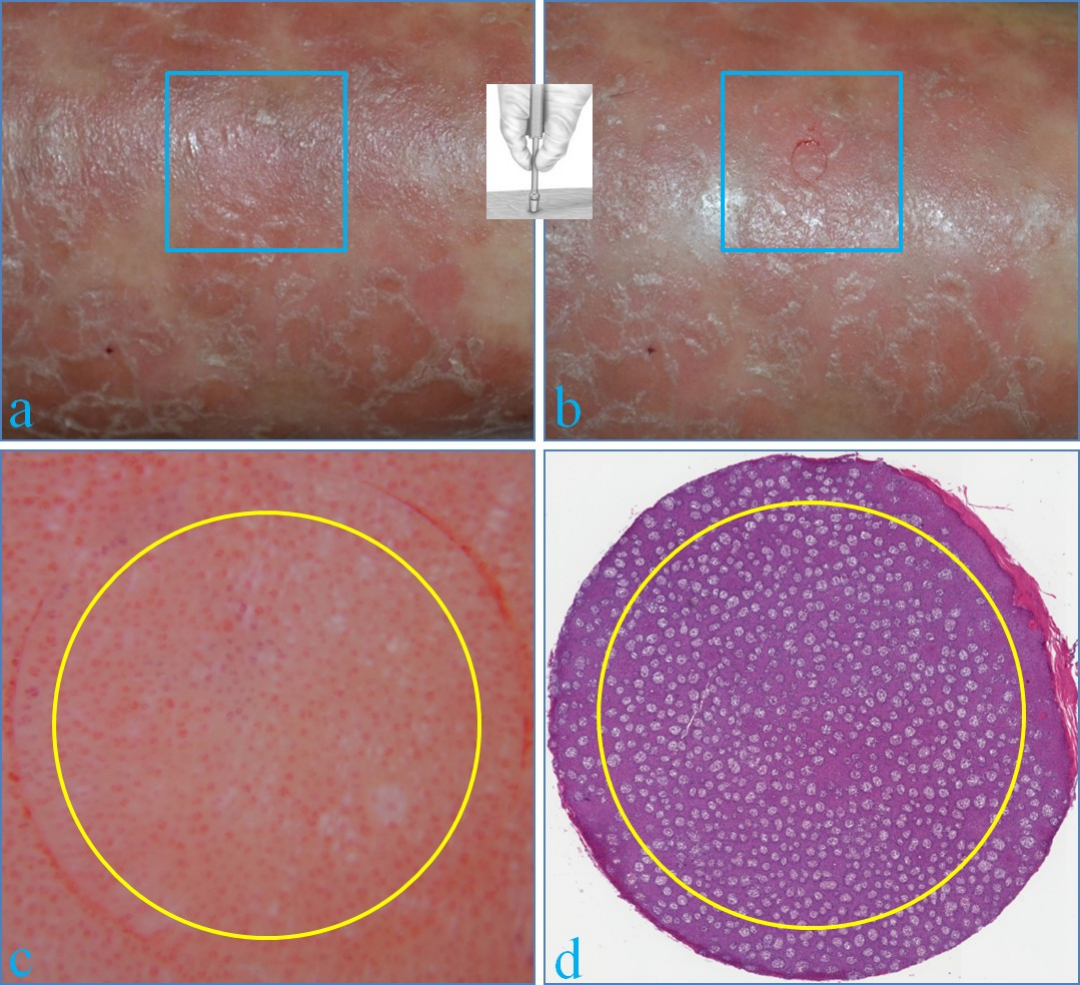
**Stable psoriatic plaque of the lower leg before (a) and after engraving a target area by gently rotating a 5-mm biopsy punch device (b).** Dermoscopy (x10) (c) and HHS (H&E stain: x100) (d) of the target area: the capillary density was evaluated in both cases in a centered 4-mm diameter area (yellow circle) by counting the number of red dots and of dermal papillae, respectively.

Twenty adult patients with stable plaque psoriasis (9 males, 11 females; median age: 46.3 years) were enrolled in the study after obtaining informed consent. Exclusion criteria were treatments with topical or systemic anti-psoriatic drugs in the last 2 or 4 weeks, respectively, as well as phototherapy/sun exposure in the previous 4 weeks, pregnancy, smoking and/or alcohol assumption, presence of microangiopathic diseases including diabetes and connective vascular diseases. Hyperkeratotic plaques were excluded as scales may hamper the dermoscopic visualization of the vascular pattern. Fifteen minutes before and during the examinations, patients stood in supine position in a temperature/humidity-controlled room. In each patient, a target area of the examined plaque previously engraved by gently rotating a 5-mm biopsy punch device, underwent dermoscopy using a x10 magnification polarized instrument (Dermlite/3Gen^TM^) connected to a digital camera, then local anesthesia with 1% mepivacaine solution, and finally skin sampling for HHS (**Fig 1A and 1B**). After paraffin embedding, consecutive 5-μm thick sections from the stratum corneum down to the superficial dermis were obtained for HHS and stained with standard Hematoxylin-Eosin. For evaluation accuracy, in all examined fields, capillary density was evaluated in a centered 4-mm diameter area, counting the number of red dots at dermoscopy (**Fig 1C**) and of dermal papillae at the dermo-epidermal junction at HHS (**Fig 1D**) by 2 different operators (AEV and FL, respectively). For each biopsy, the histological section containing the maximum count of dermal papillae was considered. Pearson’s test was performed to evaluate the correlation between the 2 methods. The study was approved by the local human research board (Catania 1). Written consent was obtained from patients and data were analyzed anonymously.

## Results

A total of 20 target lesions located on the trunk (10 cases), arms (5 cases) and thighs (5 cases) were evaluated.

The mean capillary density resulting from HHS was 50.30±9.05/mm^2^, whereas that from dermoscopy was 43.02±6.60/mm^2^. These results showed a strong correlation at Pearson’s test (r = 0.88).

## Discussion

In conjunction with epidermal hyperplasia, papillomatosis and inflammatory infiltrate, the expansion of the superficial dermal microvasculature represents one of the main histopathological changes in psoriatic skin. It consists of the presence of dilated and tortuous vessels within elongated dermal papillae, describing multiple loops in their trajectory [14]. This microvascular aspect has been evaluated by different techniques **[Table 1]** including dermoscopy, videocapillaroscopy (or capillaroscopy), fluorescence angiography, reflectance confocal microscopy (RCM) and histopathology [15].

**Table 1:** Main techniques for the evaluation of microcirculation in psoriasis.

TECHNIQUE	VISUALIZED STRUCTURES	DESCRIBED VASCULAR PATTERN
Dermoscopy	Magnified view of cutaneous structures including vascular components up to the medium dermis at low magnification (10X)	Uniformly distributed dotted red capillaries (also defined as “red dots”)
Videocapillaroscopy/Native capillaroscopy Fluorescence angiography (if contrast medium is used)	Magnified view of the cutaneous vascular structures up to the medium dermis at high magnification (100-1000X)	Twisted capillaries, with a typical glomerular appearance, homogeneously distributed throughout the entire lesion (also defined as “capillaries”, “capillary loops”, “bushy capillaries”, “tortuous capillaries”, “microvessels”, “elongated and tortuous superficial microvessels”)
Reflectance confocal microscopy	Microscopic imaging of cutaneous structures including vascular components of the superficial dermis at cellular-level resolution	Dermal papillae containing round or linear dark canalicular structures, delineated by thin walls; in living images, blood flow may be visible within them

The review of the existing literature on the different techniques to evaluate capillary density in psoriasis [4–13] has shown several critical issues **[Table 2]**. All studies differed for multiple factors including study design, methodology, and heterogeneity of results according to the instrumentation used. Also, many studies dealt with a limited sample of patients [5, 7, 11, 12]. As regards study design, the main differences consisted in non standardized exclusion criteria, with studies excluding subjects with microangiopathic disease such as diabetes and connective vascular disorders [7–10], and others not specifying any [4–6, 11–13]. Also, the site of the target plaque was variable (scalp, elbow, forearm, etc.) or unspecified [6, 7, 13]. Most studies evaluated untreated plaques, and wash out from previous treatments were quite variable [6–8, 10–12]. Methodology also varied as in some studies the instrumental evaluation was preceded by removal of scales using a lancet, a tape stripping or a keratolytic agent [4, 8, 10–12] whereas, in others, hyperkeratotic plaques were excluded *a priori* [5]. Investigational setting in most of the cases was not standardized, with few studies specifying room temperature and patient position during evaluation [4, 6–8, 10]. Also, terminology of the evaluated parameters varied from number of “capillaries” to “capillary loops”, “microvessels”, “red dots” or “dermal papillae”. The results were also quite heterogeneous as in 4 studies using videocapillaroscopy at different magnifications to count the number of capillary loops, the results ranged from a mean of ~22/mm^2^ to a mean of ~53/mm^2^ [4, 6–8, 10]. Another videocapillaroscopy study estimated the number of capillaries per “field”, but the measurement unit of the observation area was not specified [9]. As regards capillary density evaluation by RCM, considering that each dermal papilla is centred by a capillary loop, the number of capillaries likely corresponds to the number of dermal papillae. Existing studies show contrasting results, with a density ranging from a mean of 8.12/mm^2^ to a mean of ~216/mm^2 ^[5, 12, 13]. In another study that compared RCM (8 lesions) with HHS (5 lesions), the number of dermal papillae ranged from ~294/mm^2^ to~100/mm^2^, respectively [11]. In the same study, dermoscopy showed “red dots” density of 36.7/mm^2^ [11].

**Table 2:** Studies evaluating capillary density in psoriasis.

REFERENCE	TECHNIQUE	MAGNIFICATION	EVALUATED LESIONS (N)	EVALUATED PARAMETER	RESULTS	COMMENTS
Bull RH. et al, 1992^4^	Native capillaroscopy	x60-x240	22	Number of capillaries	Mean: 53.8/mm^2^	
	Fluorescence angiography	x60-x240	22	Number of capillaries	Mean: 61.3/mm^2^	
González S. et al, 1999^5^	RCM		3	Number of dermal papillae	From 6 to 9 per 250x250 μm of field of view	Resulting number of dermal papillae corresponding to 96-144/ mm^2^
						Limited sample
De Angelis R. et al, 2002^6^	Videocapillaroscopy	x100-x200	15	Number of capillary loops	Mean: ~30/mm^2^	
Hern S. et al, 2005^7^	Native capillaroscopy	Not specified	10	Number of microvessels	Mean: 4.82/mm^2^	Limited sample
Rosina P. et al, 2007^8^	Videocapillaroscopy	x200	30	Number of capillary loops	Mean: ~23/mm^2^	
Campanati A. et al, 2009^9^	Videocapillaroscopy	x60	18	Number of tortuous capillaries	Mean: 120.4 per field	Field size not specified
Rosina P. et al, 2009^10^	Videocapillaroscopy	x200	30	Number of capillary loops	Mean: ~29/mm^2^	
Wolberink EA. et al, 2011^11^	Dermoscopy	x10	8	Number of red dots	Mean: 36.7/mm^2^	Limited sample
	RCM		8	Number of dermal papillae	Mean: 293.8/mm^2^	
	Histopathology		5	Number of dermal papillae	Mean: 100.4/mm^2^	
Wolberink EA. et al, 2012^12^	RCM		6	Number of dermal papillae	Mean: ~54 per 0.5x0.5 mm of field of view	Resulting number of dermal papillae corresponding to ~216/mm^2^
						Limited sample
Hoogedoorn L. et al, 2015^13^	RCM		26	Number of dermal papillae	Mean:1812/cm^2^ (“stable” plaques) and 1996/cm^2^ (“unstable” plaques)	Resulting number of dermal papillae corresponding to 18.1/mm^2^ and 19.9/mm^2^, respectively
Present study	Dermoscopy	x10	20	Number of red dots	Mean: 43.02/mm^2^	
	HHS		20	Number of dermal papillae	Mean: 50.3/mm^2^	

Peculiarities of our work are represented by a rigorous standardization of the inclusion/exclusion criteria and methods and by the use of 2 different techniques able to provide a horizontal view of the skin, with one of them being histopathology, which represents the gold standard for skin structure evaluation. Of note, the preliminary skin engraving allowed a perfect match between the area undergoing dermoscopy and that of skin sampling for HHS. In our study, HHS showed a mean density of ~50/mm^2^ dermal papillae and the results obtained by dermoscopy were similar (~43/mm^2^). Pearson’s test demonstrated a strong correlation between the two techniques. Although capillary density resulting from dermoscopy was slightly lower compared to HHS likely due to deep-located vessels that might have gone undetected, dermoscopy, irrespective of the number of capillaries that may be observed, confirmed to be a useful non-invasive technique to evaluate the vascular pattern in psoriasis for diagnostic and research purposes (15).

An average of 50 capillaries/mm^2^ corresponds, in a common 5x5 cm psoriatic patch, to about 125.000 capillaries. These results confirm the extraordinary potential of psoriatic skin to develop such a complex and intricate vascular network. Whether the capillary density is reactive or rather plays a pathogenetic role needs to be defined. For future studies, a standardization of the patient selection and methodology, independently to the imaging technique used, is advisable.

## Supporting information

S1 Data (DOCX)
