# Supplementary material for: Evaluation of capillary density in psoriasis: An intrapatient study and literature review
Source: PLoS One. 2021 Mar 10;16(3):e0247835. doi: 10.1371/journal.pone.0247835 (PMC7946227; doi:10.1371/journal.pone.0247835)
Supplement: S1 Data — (DOCX) [file pone.0247835.s001.docx]

| Patients | Number of capillaries  (dermoscopy) | Density (dermoscopy)  mm^2^ | Number of capillaries (horizontal histopathological sections) | Density (horizontal histopathological sections)  mm^2^ |
| --- | --- | --- | --- | --- |
| 1 | 615 | 48,96 | 746 | 59,39 |
| 2 | 620 | 49,36 | 624 | 49,68 |
| 3 | 590 | 46,97 | 712 | 56,7 |
| 4 | 564 | 44,9 | 600 | 47,77 |
| 5 | 472 | 37,58 | 604 | 48,09 |
| 6 | 389 | 30,97 | 404 | 32,16 |
| 7 | 640 | 50,95 | 768 | 61,14 |
| 8 | 555 | 44,19 | 731 | 58,2 |
| 9 | 431 | 34,39 | 567 | 45,14 |
| 10 | 623 | 49,6 | 736 | 58,6 |
| 11 | 608 | 48,41 | 631 | 50,24 |
| 12 | 580 | 46,18 | 694 | 55,25 |
| 13 | 584 | 46,5 | 621 | 49,44 |
| 14 | 420 | 33,44 | 441 | 35,11 |
| 15 | 398 | 31,69 | 430 | 34,24 |
| 16 | 637 | 50,72 | 751 | 59,79 |
| 17 | 570 | 45,38 | 719 | 57,24 |
| 18 | 480 | 38,22 | 607 | 48,33 |
| 19 | 543 | 43,23 | 638 | 50,8 |
| 20 | 487 | 38,77 | 513 | 40,84 |
